# A novel adjustable automated system for inducing chronic intermittent hypoxia in mice

**DOI:** 10.1371/journal.pone.0174896

**Published:** 2017-03-31

**Authors:** Dora Polšek, Marcel Bago, Marija Živaljić, Ivana Rosenzweig, Zsombor Lacza, Srećko Gajović

**Affiliations:** 1 Croatian Institute for Brain Research, University of Zagreb School of Medicine, Zagreb, Croatia; 2 Institute of Clinical Experimental Research, Semmelweis University, Budapest, Hungary; 3 University of Physical Education, Budapest, Hungary; 4 Sleep and Brain Plasticity Centre, Department of Neuroimaging, King's College London, London, United Kingdom; 5 Sleep Disorders Centre, Guy's and St Thomas's Hospitals NHS Trust, London, United Kingdom; Tokai University, JAPAN

## Abstract

**Background:**

Sleep apnea is a chronic, widely underdiagnosed condition characterized by disruption of sleep architecture and intermittent hypoxia due to short cessations of breathing. It is a major independent risk factor for myocardial infarction, congestive heart failure and stroke as well as one of the rare modifiable risk factors for Alzheimer’s Dementia. Reliable animal disease models are needed to understand the link between sleep apnea and the various clinically linked disorders.

**New method:**

An automated system for inducing hypoxia was developed, in which the major improvement was the possibility to efficiently adjust the length and intensity of hypoxia in two different periods. The chamber used a small volume of gas allowing for fast exchanges of different oxygen levels. The mice were kept in their cages adapted with the system on the cage lid. As a proof of principle, they were exposed to a three week period of intermittent hypoxia for 8 hours a day, with 90 s intervals of 5, 7% and 21% oxygen to validate the model. Treated (n = 8) and control mice (no hypoxia, n = 7) were handled in the same manner and their hippocampal brain regions compared by histology.

**Results:**

The chamber provided a fast, reliable and precise intermittent hypoxia, without inducing noticeable side effects to the animals. The validation experiment showed that apoptotic neurons in the hippocampus were more numerous in the mice exposed to intermittent hypoxia than in the control group, in all tested hippocampal regions (cornu ammonis 1 (CA1) *P <0*.*001*; cornu ammonis 3 (CA3) *P <0*.*001*; and dentate gyrus (DG) *P = 0*.*023*). In both, control and hypoxic conditions, there was a significantly higher number of apoptotic neurons in the DG compared to the CA1 and CA3 subfields (*P <0*.*001*).

**Conclusion:**

The new design of a hypoxic chamber provides a fast, adjustable and reliable model of obstructive sleep apnea, which was validated by apoptosis of hippocampal neurons.

## Introduction

Sleep apnea is characterized by periodical cessation of breathing with a reduction in nasal airflow less than 30 percent of its normal level. It is present in three forms: central, obstructive or mixed. In central sleep apnea, the feedback mechanism in the respiratory centers of the ventrolateral medulla fails, resulting in a reduced or absent drive to breathe during sleep. The more common obstructive sleep apnea (OSA) is characterized by reduction in airflow to the lungs that occurs during sleep as a result of occlusion or narrowing of the respiratory tract at the pharyngeal level [1]. In all cases, sleep apnea leads to intermittent hypoxia, hypercapnia and subsequent reoxygenation as well as a disruption of sleep architecture. OSA has been reported to affect middle-aged and older individuals, with the prevalence recently estimated to be around 22% in men and 17% in women [2].

As obesity and ageing, the most important conditions that precipitate OSA are increasing, a significant rise in the prevalence of OSA is likely in the future [3]. It has been reported that sleep apnea is a major independent risk factor for cardiovascular diseases such as systemic and pulmonary hypertension, congestive heart failure and stroke [4] as well as myocardial infarction, cerebrovascular dysfunction and idiopathic sudden death [5]. Although association of OSA with cardiovascular and metabolic morbidity is well recognized and described, its potential role in the etiology of chronic kidney disease and its association with cancer was indicated only recently [6], as well as its potential role in brain injury leading to neurodegenerative disorders [7]. OSA has an important effect on cognitive function, in particular in episodic verbal learning, memory, cognitive flexibility and mental processing speed and more. It is also one of the rare modifiable risk factors for Alzheimer’s disease. The patients with Alzheimer’s disease have a five times higher chance of presenting with OSA than cognitively non-impaired individuals of similar age [8].

On the contrary, mild sleep apnea can have a beneficial preconditioning effect on the body in the context of cerebrovascular disease [9]. There have been numerous animal studies showing both beneficial and detrimental consequences in relation to the pattern and depth of hypoxia exposure. In addition, clinical research has underlined the importance of including severity of intermittent hypoxia as a relevant factor in making sense of large quantities of sometimes contradictory data [10].

An animal model, which would recreate the conditions of sleep apnea as closely as possible, is needed to clarify both the consequences and potential therapeutic strategies. Among current systems available for exposing animals to hypoxia, most are manually controlled, expensive or bulky. Although designed to provide hypoxia, their major disadvantage in modelling the obstructive sleep apnea is that they are too slow to reach set oxygen levels in the limited time period of low oxygen exposure and are therefore inadequately precise for defining experimental protocols that allow studying subtle differences between mild, moderate and severe sleep apnea. Finally, some systems require exposing the mice to a new chamber environment, thus contributing to the stress levels for the animals.

Therefore, the aim of our study was to develop an automated system that could mimic the gas exchanges and create intermittent hypoxia similar to those occurring in patients during sleep. Moreover, it was desirable to allow maximal adaptability of experimental protocol by controlling different oxygen levels, their duration, and fast exchanges. As the periods of hypoxia are short but repetitive, it was important to develop a system that is fast to reach the set value of oxygen in the chamber, permitting the animal exposure to very short periods of hypoxia. To minimize animal stress, the system was made to be compatible with an existing animal housing cage to reduce the effect of changing the environment for the experimental animals. The system had to meet the needs of noise, humidity and gas flow control and assure the reliability of the experimental protocol.

Given the vulnerability of the region to a range of pathologies and considering the consistency of the presentation of neurocognitive impairments in sleep apnea [11, 12], neuronal apoptosis in the hippocampus was evaluated in this study in the context of chronic intermittent hypoxia. The hippocampus was selected for the validation of our new mouse model because clinical research points to the degeneration of the same anatomical regions in the patients suffering from obstructive sleep apnea as it does in mice [9, 13].

## Materials and methods

### The mouse model of intermittent hypoxia

The mice were bred and housed in standard animal cages in the animal facility of the Croatian Institute for Brain Research, University of Zagreb School of Medicine. Light-dark cycle was set to 12 h-12 h beginning at 7 h and 19 h respectively. Food and water was available ad libitum in the cages as well as during the intermittent hypoxia treatment. The experiments were approved by the Ethical Committee of the University of Zagreb School of Medicine (permit number: 380-59-10106-14-55/230).

Male mice of C57BL6 inbred strain aged 85 ± 20 days were randomly allocated into two groups, animals exposed to intermittent hypoxia (IH, n = 8) and a control group (CTRL, n = 7). CTRL mice were handled, and housed in a manner consistent with that of the IH mice, most importantly being in the same size cages and in the same room as the treated animals, but not being exposed to hypoxia. This allowed controlling for the effects of the handling-stress, noise and disturbance being same as in the intervention group.

The IH mice were exposed to a 21 day protocol for 8 hours per day of 90 s periods of 5.7% of oxygen followed by 90 s period of 21% oxygen. The flow of gases was 50 L/min with the set pressures of 2 bars of nitrogen and 1.2 bars of air. The protocol was administered using a new designed system explained in this article.

### Brain histology

After the 21-day study period, mice were anaesthetized by administration of 0.5 g/Kg tribromoethanol (Avertin, Sigma-Aldrich, USA) and transcardially perfused with 10 ml phosphate buffered saline (PBS) followed by 10 ml of 4% paraformaldehyde solution, and the brains isolated. The isolated brains were kept for 24 h immersed in 4% PFA, kept in PBS with 0.05% sodium azide and transferred to 30% sucrose solution 3 days before cutting.

The brains were embedded into TissueTek fluid (Sakura, Netherlands), mounted on the platform, frozen with dry ice and cut using a microtome (HM 430 TermoFisher, USA) in 35 μm thick coronal sections. The glass slides with tissue sections were immersed in 0.05% Nissl stain solution mixed with few drops of 10% acetic acid for 30 minutes, rinsed in distilled water and dehydrated in ascending concentrations of ethyl alcohol. Samples were cleared in xylene and mounted.

For every mouse 4 representative coronal sections containing the hippocampal formation were selected using the Brain Allen Mouse Atlas as a reference (http://mouse.brain-map.org) and the regions of interest Cornu ammonis 1 (CA1), Cornu ammonis 3 (CA3) and the dentate gyrus (DG) were identified. In order to eliminate the possibility of a systematic error due to a difference in area of selected regions of interest, same sizes of examined areas were selected.

The apoptotic neurons were stereologically counted within the 280 μm x 280 μm counting frame for the regions of interest on a Nikon YS100 microscope, 40x magnification, and subsequently numbers of apoptotic neurons per mm^3^ were calculated. The experimenter was blinded to the mouse label and group affiliation.

All data was processed and plotted using GraphPad Prism 6 software, tested for normality of distribution using Shapiro-Wilk test and analyzed using ANOVA with Holm-Sidak’s multiple comparison test. Statistical significance was defined as lower than *P<0*.*05*.

## Results

### Intermittent hypoxia system design

The system was designed to address the requirements for adjustable, fast and precise experimental protocols of intermittent hypoxia allowing the improved modelling of sleep apnea (Table 1).

**Table 1:** Requirements and technical solution applied in the new intermittent hypoxia system.

Requirements	Technical solutions
Maximum adjustability in the design of the experimental protocols in terms of hypoxia levels and exposure times	Controller enabling setting two different oxygen levels and two time periods
Fast transition between different oxygen levels to allow short hypoxia periods	Dual sensor input to the controller board
	Mixing antechamber with oxygen sensor
	Small volume
	High gas flow
Precise oxygen control in the chamber	Mixing antechamber with oxygen sensor
	Dual-sensor input to the controller board
	Use of Techniplast GM500 cage with patented sealing design
Evenly distributed gas flow throughout the chamber	Modification of Techniplast GM500 cage with patented ergonomic design
Minimizing animal suffering	System compatible with the housing cage
	Humidifier
	Muffler system
	Standard housing space for animals

**Fig 1:**
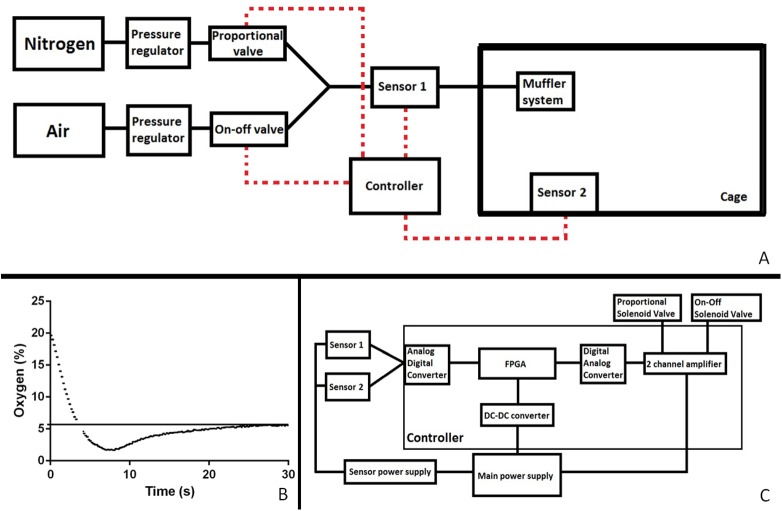
**A block diagram of the complete setup (A), the speed and pattern of lowering the oxygen to a set concentration (B), a block diagram detailing the controller and the dual sensor interface (C).** The solid line denotes airflow and the dashed line the electrical connections. FPGA, field programmable gate array; DC-DC digital to digital converter.

A block diagram of the entire setup is represented by Fig 1. Air supplied through a compressor (SAI2088, SMC, USA) and bottled nitrogen (99,996%, Messer, Croatia) entered the system via separate inlets. Rough pressures were manually defined on the manometers of the compressor and the bottle respectively. Upon entering the system, both tubes were connected to more precise manometers and finer pressure regulators, where the pressure could be manually fine-tuned. The air tube was then connected to two valves, an on- off (D885, M&M International, USA) and a small proportional valve (PVQ13, SMC, USA). The nitrogen tube was connected to a bigger proportional valve (PVQ31, SMC, USA). The tubes converged and another manometer was placed to check the pressure of the mix. Subsequently, the tubes diverged, one arm led to a mixing antechamber (10mm x 10mm x 500mm) mounted with its own oxygen sensor (O2-A2, Alphasense, UK), and the other arm passed through a water humidifier. The two arms converged to enter the hypoxia chamber, through the inlet originally designed to fit with the individually ventilated cage (IVC) system for the cage. A muffler system was mounted on the inside of the lid, augmenting the gas entrance area by 4 times. A second oxygen sensor (O2-A2, Alphasense, UK) was mounted from the inside of the cage lid and monitored the actual gas mix in the cage. The outlet from the cage, designed for the IVC system, remained open throughout the protocol to prevent any pressure from rising in the chamber.

**Fig 2:**
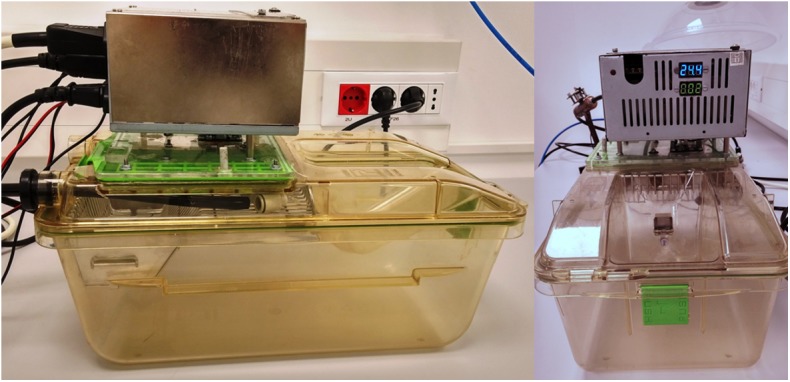
A photograph of how the mouse cage lid was modified to fit the regulating apparatus of the system.

To address the demand of small volume and minimizing the stress of changed environment to the animals the system was developed to be compatible with a sealed mouse cage 391 x 199 x 160mm (GM500 Tecniplast, Italy, Patent No. ZL200610076641.0; 8.037.847; 1719406), making it possible to apply experimental hypoxia without the need to move the mice from their original housing. A lid of the mouse cage was modified to fit the regulating apparatus of the system (Fig 2). The sealing of the cage and the ergonomically-designed flow of gas was primarily intended for individually ventilated animal housing and patented by Tecniplast, but through our adaptation solved the necessity for a sealed volume and evenly distributed gas flow in the hypoxia chamber.

The controller consisted of a field programmable gate array (FPGA) (Basys2, Digilent, USA) card with a LED display and a user interface mounted on the cage lid. The interface used 4 buttons where the percentage of oxygen and the time of maintaining the set oxygen level could be specified for two distinct time periods and two distinct oxygen levels. The buttons allowed changing the parameter displayed on the LED display, setting the parameter value, saving the parameter setting, and starting the protocol.

A computer program in VHSIC Hardware Description Language (VHDL) was written for the FPGA within the regulating device to use the input from both sensors and the set parameters to calculate the current needed to control the diameter of the opening of 2 proportional valves and a third on-off valve (S1 Computer Code). The program simulated a proportional- integral-derivative controller. The FPGA was fitted with an analogue- digital and digital- analogue converters to be able to process the signals from the sensors and to the valves (Fig 1C). The program used the antechamber sensor input to modify the gas mix rapidly, while the cage sensor input signaled to turn off the gas flow when the set oxygen level was reached. This also allowed minimizing the use of nitrogen and air.

The compressor supplying air was connected to two separately controlled valves to lower the risk of the system undershooting. Namely, we have noticed that in experimental protocols using low levels of oxygen, before some systems reach the set level there is a transitory drop in oxygen below the set level. This risk of transitory undershoot can have a catastrophic impact on the experimental animals, sometimes suffocating or unacceptably injuring them, but as well significantly influence the experimental results. For this reason, we decided to add a small proportional valve that would continuously stay open, minimizing the length and the effect of the undershoot (Fig 1B). A bigger on-off valve would nonetheless permit the fast and precise elevation of oxygen level when needed.

The muffler system was designed to slow down the flow of gas to minimize the stress of drought, noise and drying to the animals by increasing the entrance area of gas by 4 times of the original inlet.

The hypoxia chamber developed in our laboratory has been submitted for a patent, which is currently pending (GB 16602071.1).

### Intermittent hypoxia was achieved by the automated system

The intermittent hypoxia system allowed various complex hypoxia exposure patterns. The system enabled defining two different oxygen levels and two time period lengths.

The system was fast to reach the set parameters, with a mild transitory undershoot. In our experiment, when changing from atmospheric (21%) to hypoxic (5.7%) levels the system reached hypoxia in less than 5 seconds and stabilized the oxygen level to 5.7% in 27 seconds. The undershooting was also short, lasting approximately 12 seconds with a nadir of 2% of oxygen (Fig 1B).

The system was also minimally stressful for the animals, as they were able to stay in their original housing cages, with humidity changes, airflow and noise kept at minimum levels. Although the animals were more immobile and quiet then usual while the hypoxia was ongoing, they showed no signs of uneasiness or obvious effects during or after hypoxia.

### Intermittent hypoxia induces neuronal apoptosis in the hippocampus

To validate the intermittent hypoxia system, the animals were subjected to a protocol lasting 21 days and assessed for the effect of the hypoxic microenvironment on hippocampal neurons to verify whether the expected biological effects will be achieved. Neuronal apoptosis was taken as a hallmark consequence of intermittent hypoxia. A similar protocol of intermittent hypoxia using Oxycycler model A44XO (Reming Bioinstruments, USA) showed a sevenfold increase of TUNEL stained neuronal apoptosis in the CA1 region of the rat hippocampus after acute hypoxia exposure and a smaller, but statistically significant difference after 14 days of chronic intermittent hypoxia [13].

**Fig 3:**
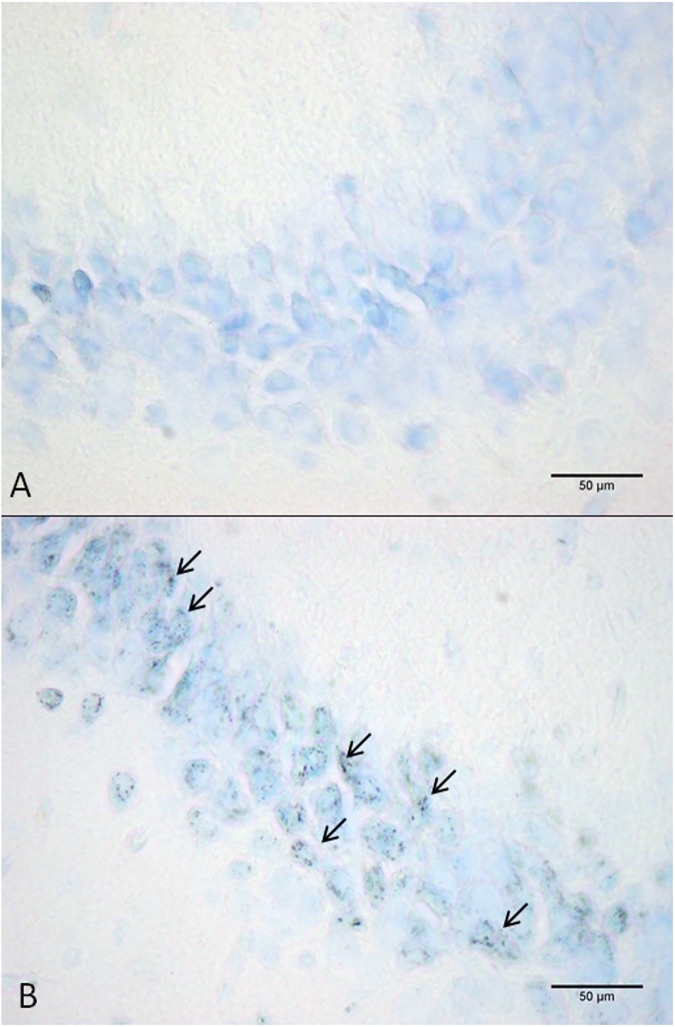
**Representative images of neuronal apoptosis in the hippocampal region cornu ammonis 3 (CA3) of mouse from the control group (A) and intermittent hypoxia group (B).** The arrows show apoptotic neurons.

**Fig 4:**
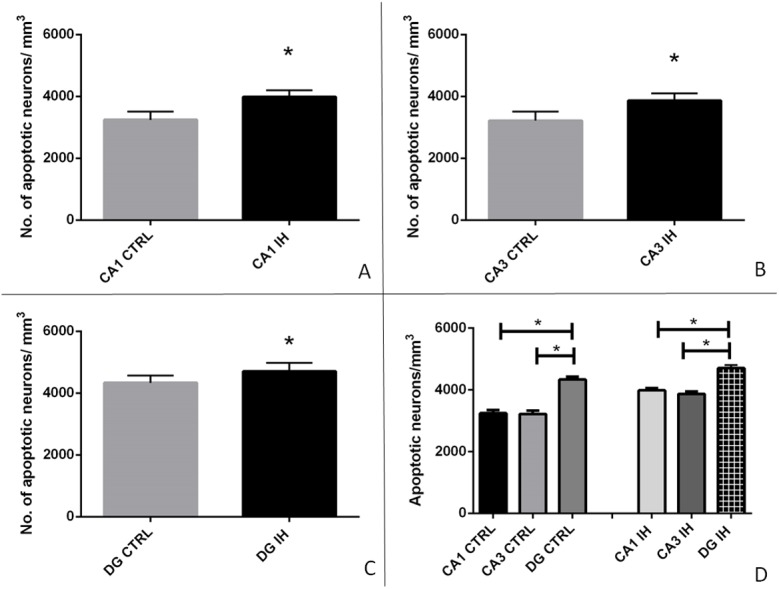
**Apoptotic neurons per mm^3^ of tissue in the control group (CTRL) compared to the intermittent hypoxia group (IH) in cornu ammonis 1(CA1) subfield (*P< 0*.*001*) (A), cornu ammonis 3 (CA3) subfield (*P = 0*.*001*) (B), and the dentate gyrus (DG) (*P = 0*.*023*) (C) shown as columns representing means and standard deviations.** The number of apoptotic neurons differed significantly in the DG IH group opposed to CA1 IH (*P<0*,*001*), and CA3 IH (*P<0*,*001*), as well as in the DG CTRL group opposed to CA1 CTRL (*P<0*,*001*), and CA3 CTRL (*P<0*,*001*) (**D**). Asterisks denote statistically significant differences.

The apoptotic neurons were determined by hyperchromia, condensation and fragmentation of chromatin, shrinking of neuron’s soma (Fig 3). By quantifying the number of apoptotic neurons in the hippocampal subfields of the exposed (n = 8) and the control groups (n = 7), we have shown statistically significant apoptosis in all three examined subfields of the hippocampus, the CA1, CA3 and DG (Fig 4A, 4B and 4C). When the means of treated and control mice were compared the increase of apoptosis in hippocampus was around 30%, which is in compliance with the existing literature for chronic IH exposure at a similar time point [13, 14], and confirms the validity of the intermittent hypoxia system applied.

The number of apoptotic neurons differed significantly in the DG of IH group opposed to CA1 IH (*P<0*,*001*) and CA3 IH (*P<0*,*001*). In addition, the number of apoptotic neurons in the DG CTRL group differed significantly opposed to CA1 CTRL (*P<0*, *001*) and CA3 CTRL (*P<0*,*001*) implicating the DG as the most dynamic region of hippocampus. No differences were found between the number of apoptotic neurons in CA1 and CA3 regions of IH mice or CTRL mice (Fig 4D).

## Discussion

### Approaches to modeling intermittent hypoxia

The intermittent hypoxia system for laboratory mice, intended to model human sleep apnea was designed, applied and verified in this study. The major improvements in relation to previous solutions was that (A) the system was fast to reach set oxygen levels with the dual sensor input to the controller improving precision of control and minimizing the time lag between set and actual parameters in the chamber, and (B) it was compact and compatible with an individually ventilated mouse cage allowing controlled gas flow and appropriate sealing.

Most non- commercial systems for inducing hypoxia incorporate a Plexiglas chamber with limited control of gas mixing and sealing, a time-regulated opening and closing of solenoid valves and a sensor for monitoring the gas in the chamber, but with no backward loop to the valves [15–18]. Most of them are voluminous cabinets, which could host many animals, but the ability of fast exchange of gasses in such volume (the requirement of intermittent hypoxia protocol is 90 seconds) remains questionable. On the other hand, some of them are very small, being able to contain only a single animal per box, prolonging and complicating the experiment [19].

The novelty of our system is the control of the solenoid valves using input from two sensors. An antechamber with an oxygen sensor is used to pre-mix the gases to a set value while another sensor monitors the actual oxygen percentage in the hypoxia chamber. Most developed systems monitor the percentage of oxygen in the chamber and thus are subjected to a time lag between set parameters and the actual chamber conditions, which in our experience can be quite significant, especially in very short hypoxia exposures.

Another approach to modelling intermittent hypoxia is using a commercial oxygen controller that is made to be compatible with an incubator where a cage or several cages can be placed within [14]. These systems are very practical, being almost completely automated throughout the several week periods. However, systems with big volumes can be slow to reach set parameters in short periods of time. For illustration, Xu et al. used a chamber with dimensions of 30x20x20 inches (V = 12000 in^3^; 196.6L) while our chamber measures 15.40x7.83x6.30 inches (V = 759, 66 inches^3^; 12.4 L) [20]. The gas flow to the chamber used was roughly the same, measuring 60 l/min in the experiment by Xu et al. and 50L/min in our experiment.

There have been several other comparable models of intermittent hypoxia, such as a system described by Li et al. where the animal chamber is inserted into a larger hypoxia box and moved out into ambient air in relation to the period of exposure [21]. In an original solution by Tagaito et al. a bell-shaped chamber floating on water was designed that enabled to monitor EEG and EMG from the exposed animal during the hypoxic exposure [22]. In our opinion, although inventive and important, these designs are limited in providing a high throughput, reproducible and easily available animal model needed for large scale experiments.

### Intermittent hypoxia induces neuronal apoptosis in the hippocampus

The biological validation of the system showed a significant increase of apoptosis in the hippocampus. This finding is in partial contradiction to previous findings [14, 23], where the authors established an increase in apoptosis in the CA1 subfield of the rat hippocampus, but found no significant increase in apoptosis in the CA3 area. In previous studies the dentate gyrus was implicated as the location of hippocampal neurogenesis following intermittent hypoxia, showing cells co-positive for neurofilament and BrDU [24]. Recently, lower numbers of neurons after intermittent hypoxia have been shown in the mouse [25] and rat DG as well [26].

The reasons for the diverging of the finding of CA3 neuronal apoptosis in our results might have twofold explanation. The intermittent hypoxia studies dealing with differences of the sensitivity of hippocampal subfields have been done on rats [14, 23], while our model vas validated studying mice. Secondly, the CA1 subfield has been shown to be more susceptible to hypoxic damage then CA3 subfield in the models using large- volume chambers. We propose that our system was faster to reach the set hypoxic parameters and more precise in keeping the parameters stable and thus resulted in a more severe hypoxia, possibly thus affecting the less sensitive CA3 subfield.

The presented intermittent hypoxia system is intended to model human sleep apnea. Still the relevance of the presented animal study has limitations. The young healthy mice were used in the current study, while the patients with obstructive sleep apnea are usually elderly people with several associated diseases. Moreover, although the 8 hour protocol was administered during the day when mice predominantly sleep with intention to mimic the sleep fragmentation, mice sleep in phases with arousals every 15 to 20 minutes, which is uncommon in humans.

## Conclusion

The design of a new hypoxic chamber provided a fast, adjustable and compact model of obstructive sleep apnea, which was validated by showing apoptosis of neurons in the hippocampus.

## Supporting information

S1 Computer Code Comprehensive collection of computer codes written in VHSIC Hardware Description Language (VHDL) for the programming of various parameters of the hypoxic chamber regulators. (ZIP)
